# The Complex Relationship between Diabetic Retinopathy and High-Mobility Group Box: A Review of Molecular Pathways and Therapeutic Strategies

**DOI:** 10.3390/antiox9080666

**Published:** 2020-07-26

**Authors:** Marcella Nebbioso, Alessandro Lambiase, Marta Armentano, Giosuè Tucciarone, Vincenza Bonfiglio, Rocco Plateroti, Ludovico Alisi

**Affiliations:** 1 Department of Sense Organs, Faculty of Medicine and Odontology, Policlinico Umberto I, Sapienza University of Rome, p. le A. Moro 5, 00185 Rome, Italy; marcella.nebbioso@uniroma1.it (M.N.); marta.armentano@uniroma1.it (M.A.); giosue.tucciarone@uniroma1.it (G.T.); rocco.plateroti@uniroma1.it (R.P.); ludovico.alisi@uniroma1.it (L.A.); 2 Department of Ophthalmology, University of Catania, Via S. Sofia 76, 95100 Catania, Italy; vincenzamariaelena.bonfiglio@unipa.it

**Keywords:** antioxidants, diabetes mellitus, diabetic retinopathy, free radicals, high-mobility group box 1 (HMGB1), inflammatory pathways, novel therapies, oxidative stress

## Abstract

High-mobility group box 1 (HMGB1) is a protein that is part of a larger family of non-histone nuclear proteins. HMGB1 is a ubiquitary protein with different isoforms, linked to numerous physiological and pathological pathways. HMGB1 is involved in cytokine and chemokine release, leukocyte activation and migration, tumorigenesis, neoangiogenesis, and the activation of several inflammatory pathways. HMGB1 is, in fact, responsible for the trigger, among others, of nuclear factor-κB (NF-κB), tumor necrosis factor-α (TNF-α), toll-like receptor-4 (TLR-4), and vascular endothelial growth factor (VEGF) pathways. Diabetic retinopathy (DR) is a common complication of diabetes mellitus (DM) that is rapidly growing in number. DR is an inflammatory disease caused by hyperglycemia, which determines the accumulation of oxidative stress and cell damage, which ultimately leads to hypoxia and neovascularization. Recent evidence has shown that hyperglycemia is responsible for the hyperexpression of HMGB1. This protein activates numerous pathways that cause the development of DR, and HMGB1 levels are constantly increased in diabetic retinas in both proliferative and non-proliferative stages of the disease. Several molecules, such as glycyrrhizin (GA), have proven effective in reducing diabetic damage to the retina through the inhibition of HMGB1. The main focus of this review is the growing amount of evidence linking HMGB1 and DR as well as the new therapeutic strategies involving this protein.

## 1. Introduction to Diabetic Retinopathy (DR)

**Figure 1:**
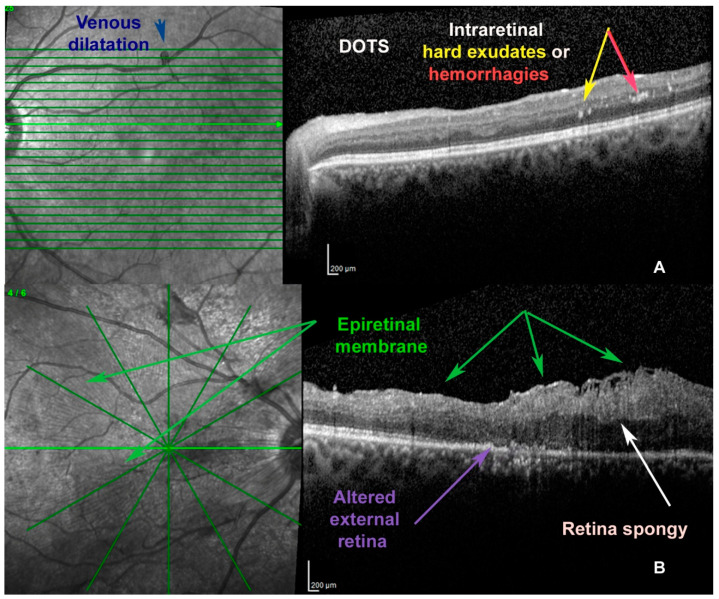
Spectral-domain optical coherence tomography exam of two patients. Patient (**A**). Initial diabetic retinopathy (DR) characterized by typical vascular and retinal abnormalities: microaneurysms, hemorrhages (hyperreflective dots), hard exudates (hyperreflective dots), and venous dilatation (blue arrow). Patient (**B**). Preproliferating ischemic-exudative DR to the posterior pole with epiretinal membrane (green arrows), retina spongy (white arrow), and altered layers of photoreceptors and retinal pigment epithelium (external retina).

**Figure 2:**
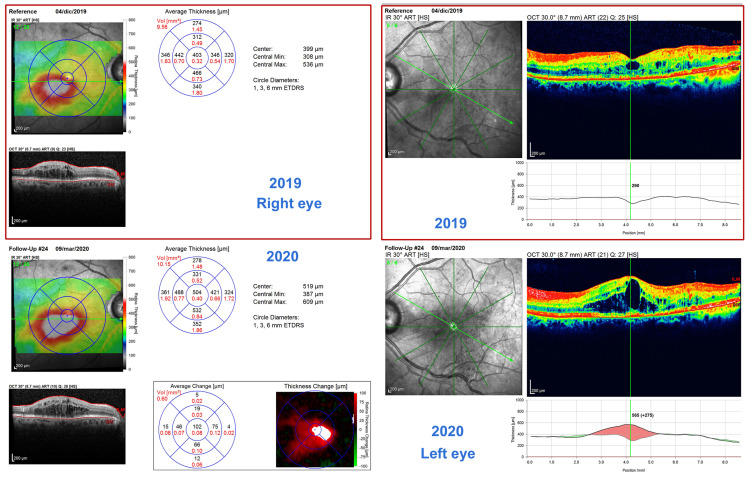
Spectral-domain optical coherence tomography (SD-OCT) of a diabetic patient right and left eye. The progression of the disease toward the stage of proliferative diabetic retinopathy (PDR) is defined by the development of persistent/chronic macular edema with flower petal cysts, important retinal and vitreous hemorrhages, marked fibrovascular proliferation, tractive retinal detachment, and, finally, vitreoretinal neovascularization with retinal detachment. SD-OCT exam: above 04 December 2019 and under 09 March 2020.

Diabetes mellitus (DM) is a well-known metabolic disease that causes numerous chronic complications. To this day, the number of patients affected by type one and type two DM is estimated to be 463 million. This number will rise to 700 million by 2045 (around 10.9% of the global population) [1]. The most common complications, such as diabetic retinopathy (DR), are caused by microvascular damages. Nowadays, the number of patients affected by DR is calculated as around 93 million [2]. These numbers place DR as the fifth most common cause of severe visual impairment in the world [3]. From a clinical point of view, DR is characterized by typical vascular and macular abnormalities. In non-proliferative DR (N-PDR), the most common findings are microaneurysms, cotton wool spots, hemorrhages, hard exudates, and venous dilatation. The progression toward the stage of PDR is defined by the development of neovascularization that may lead to retinal and vitreous hemorrhages, fibrovascular proliferation, and tractive retinal detachment. Other complications of DR are neovascular glaucoma, steaming from iris neovascularization, and macular edema (Figure 1 and Figure 2) [4,5].

The pathogenesis of DR is an extremely complex mechanism that involves numerous biochemical and inflammatory pathways triggered by long exposition to hyperglycemia. The development of DR is characterized by the concomitant participation of vascular endothelial dysfunction, pericyte loss, and neurodegeneration, which ultimately leads to hypoxia and neovascularization [6]. Interestingly, neuronal degeneration appears to precede vascular disease and develop as an independent mechanism [7].

The persistence of high levels of blood glucose is determinant for the activation of inflammatory mechanisms, the enhancement of oxidative stress, and, consequently, the production of advanced glycation end-products (AGEs) [4]. The inflammation determines the local accumulation of cytokines, such as vascular endothelial growth factor (VEGF), tumor necrosis factor-alpha (TNF-⍺), and inducible nitric oxide synthase (iNOS), that favor the establishment of hypoxia in the diabetic retina [8]. Inflammation leads also to the accumulation of chemokines and adhesion molecules such as intercellular adhesion molecule-1 (ICAM-1). This mechanism causes the migration of leukocytes towards the retinal endothelium, increased vascular permeability, and the breakdown of the blood–retinal barrier (BRB) that ultimately leads to edema [9].

Hyperglycemia determines the production of AGEs. The binding of AGEs to their receptors (RAGE) allows for the activation of nuclear factor-κB (NF-*κ*B) pathways, which ultimately leads to the production of reactive oxygen species (ROS) and the reduction of antioxidant defense systems [10]. Oxidative stress conducts to the metabolic memory phenomenon in mitochondria. This phenomenon is deemed responsible for the persistence of vascular damage, even when glycemic control is perfectly achieved [11]. Moreover, hyperglycemia determines the production of sorbitol through the activation of the polyol pathway. High volumes of sorbitol lead to the depletion of reduced glutathione (GSH) and the accumulation of ROS [12].

Hyperglycemia is also able to activate different isoforms of protein kinase C (PKC). This enzyme participates in the retinal vascular damage through its efficiency in the induction of nicotinamide adenine dinucleotide phosphate oxidase (NOX). The resulting O_2_^−^ contributes to the worsening of endothelial dysfunction [13,14]. PKC is also involved in the increase of endothelial cell death and pericyte loss via the accumulation of oxidative and nitrosative products, contributing to the development of microaneurysms and the recruitment of leukocytes [15].

Another pathway heavily affected by chronic exposition to hyperglycemia is the hexosamine pathway. The alternative cycle to glycolysis is needed to convert the excess of fructose 6-phosphate that cannot be metabolized by classic glycolysis. This leads to the production of *N*-acetyl glucosamine and the overexpression of transforming growth factor-β1 (TGF-β1) and plasminogen activator inhibitor-1 (PAI-1), increasing the apoptotic rate of endothelial cells (EC) and pericytes [16,17]. Moreover, AGE accumulation activates the hexosamine pathway, determining the production of angiopoietin-2 and the development of neovascularization [18,19]. Lastly, it has been recently demonstrated that the activation of the innate immune response facilitates the development of inflammation and therefore DR. Specifically, concentrations of toll-like receptor (TLR)−4 and −2 as well as their downstream inflammatory cytokines TNF-α, interleukin (IL)-1β, and interferon (IFN)-β were found to be significantly increased in murine models of DM [20,21].

**Scheme 1:**
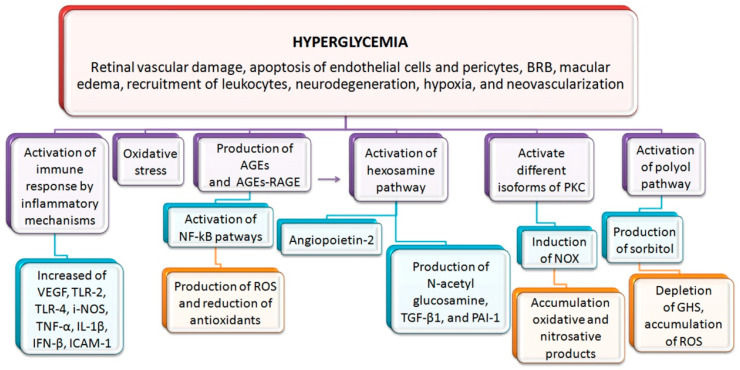
Predominant biochemical alterations in diabetes mellitus (DM) patients and related dysfunctions caused by hyperglycemia. HMGB1: high-mobility group box 1; DR: diabetic retinopathy; BRB: blood–retinal barrier; AGEs: advanced glycation end-products; RAGE: receptors for AGEs; PKC: protein kinase C; NF-κB: nuclear factor-κB; TGF-β1: transforming growth factor-β1; PAI-1: plasminogen activator inhibitor-1; NOX: nicotinamide adenine dinucleotide phosphate oxidase; VEGF: vascular endothelial growth factor; TLR: toll-like receptor; iNOS: inducible nitric oxide synthase; TNF-α: tumor necrosis factor-α; IL-1β: interleukin-1β; IFN-β: interferon-β; ICAM-1: intercellular adhesion molecule-1; ROS: reactive oxygen species; GSH: reduced glutathione.

Current therapeutic approaches, such as anti-angiogenic agents or corticosteroids intravitreal injections and laser therapy, only target the manifestations of DR. The complexity of the metabolic pathways activated during DR shows how single-target therapies have limited success [22,23,24]. The lack of preventive treatments and the increasing number of patients show the need for the development of new specific agents targeting the metabolic pathways that lead to DR (Scheme 1).

## 2. Introduction to High-Mobility Group Box 1 (HMGB1)

High-mobility group (HMG) proteins are a group of non-histone nuclear proteins discovered in 1973 in the calf thymus, including three families, named HMGB, HMGN, and HMGA [25]. This group of proteins owes their name to their high electrophoretic mobility [26]. The high-mobility group box (HMGB) family contains several different proteins unified by the constant presence of at least one HMGB. The most studied and ubiquitary protein of this family is HMGB1 [27]. HMGB1 is an evolutionary conserved chromatin-binding protein composed of 215 amino acids and characterized by two DNA binding domains named Box-A and Box-B and a C-terminal acidic domain [28]. Initially considered a nuclear protein, HMGB1 has subsequently shown a cytosolic location inside several cell organelles and structures, such as mitochondria and the cellular membrane as well as extracellular space [29].

HMGB1 may be present in a reduced, oxidized, or disulfide form. Its actions appear to be largely dependent on the redox state [30]. The reduced form is characterized by the reduction of specific cysteine residues. In this configuration, HMGB1 can recruit leukocyte independently from the release of cytokines or chemokines [31]. The oxidized configuration determines the loss of the immunogenic properties of HMGB1 [32]. Lastly, the disulfide form activates the NF-κB inflammatory pathway, determining the production of IL- 6, -8, and TNF-⍺ [31].

**Figure 3:**
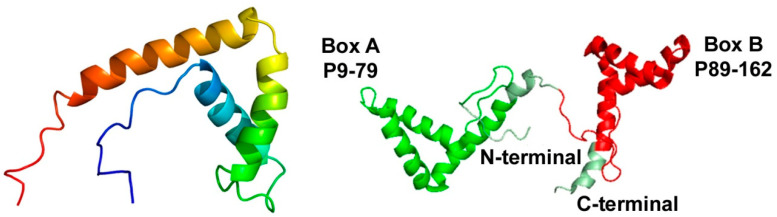
Structure of high-mobility group box 1 (HMGB1) protein. HMGB1 is a protein consisting of 216 residues, 30 kD, highly conserved among mammals. The protein contains three alpha-helices, connected together by loops. It consists of two homologous DNA-binding domains of the HMG-box type: Box A (and Box B) and segment C-terminal, a negatively charged “tail”. There are two nuclear localization sequences. Green marks the linker and terminal regions of HMGB1. Box A (P9–79) is colored in green and Box B (P89–162) in red.

HMGB1, due to its ubiquitous location in the cell, performs numerous activities (Figure 3).

Inside the nucleus, HMGB1 controls chromatin stability and replication, nucleosome release from damaged cells, gene recombination, and transcription, DNA repair, and replication [27,33].

Cytosolic HMGB1 is usually secondary to the shuttling of nuclear HMGB1 in response to hypoxia, chemokines, cytokines, and ROS. In the cytosol, HMGB1 acts as a positive regulator of autophagia [34]. HMGB1 expression on the surface of cellular membranes is responsible for the activation of innate immunity and mediates cellular adhesion [35,36].

Extracellular HMGB1 is involved in numerous activities such as the regulation of T-cells [37], stem cells [38], and neoplastic cell differentiation [39]. This protein is also involved in the management of the inflammatory response, through the activation of numerous different immune cells [40,41], and the promotion of cytokine release [42,43]. HMGB1’s extracellular functions consist of cellular proliferation [44] and migration [45], including vascular growth during inflammatory or neoplastic diseases and tissue repair [46,47]. During the inflammatory response, HMGB1 is secreted by macrophages, platelets, EC, and monocytes, as well as necrotic or damaged cells [48]. Disulfide HMGB1 binds together with myeloid differentiation factor-2 and TLR-4, determining the formation of a complex that triggers the inflammatory response [49,50]. In addition, HMGB1 deficient cellular lines show a reduced capacity to induce cytokines [51]. The binding of HMGB1 to RAGEs determines the formation of a complex responsible for the activation, among others, of NF-κB, phosphatidylinositol 3-kinase (PI3K)/PKB, mitogen-activated protein kinase (MAPK), and TNF-α pathways [52,53,54]. Thus, HMGB1 is involved in myriad diseases, such as hypoxia-induced injury [55], microglial damage and neuroinflammation [56], vascular barrier damage [57], and inflammatory heart diseases [58]. Moreover, ROS, through the activation of the NF-κB pathway, are responsible for the passive and active secretion of HMGB1 in monocytes and macrophages [59]. HMGB1 is recognized to be a direct angiogenic molecule as it induces a pro-angiogenic phenotype in EC [60,61]. It can, moreover, stimulate angiogenesis through the activation of the MAPK/extracellular signal-regulated kinase (ERK) 1/2 pathway. The bond between HMGB1 and RAGE results in the stimulation of NF-κB signaling in leukocytes, which leads to the production of proinflammatory and angiogenic molecules [62]. HMGB1 in conjunction with TLR-4 can influence the development of neovasis in proliferative and metabolic diseases [63,64]. Moreover, it has been demonstrated that HMGB1 can mediate angiogenesis through the activation of hypoxia-induced factor-1α (HIF-1α) [65].

In conclusion, HMGB1 shows a wide range of interactions in both physiological and pathological mechanisms. The next section of the review will focus on the growing amount of evidence linking HMGB1 expression and the development of DR. The main focus of this review is the growing amount of evidence linking HMGB1 and DR as well as the new therapeutic strategies involving this protein.

## 3. HMGB1 and DR

**Scheme 2:**
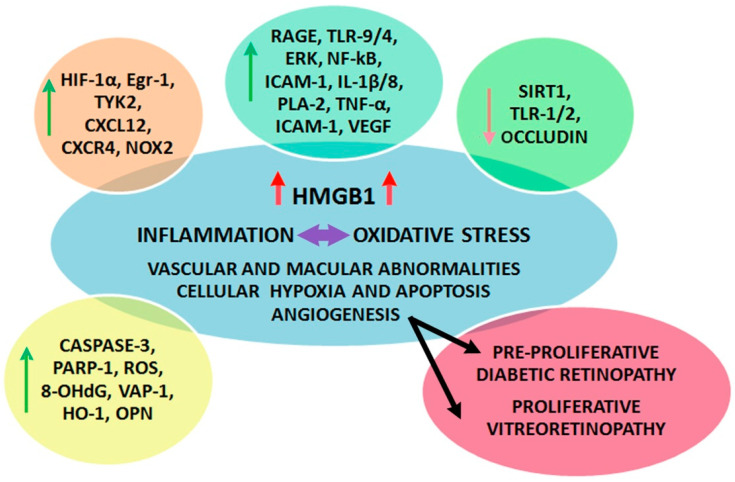
High-mobility group box 1 (HMGB1) levels increased in diabetic retinopathy (DR). HMGB1 promotes angiogenesis directly and indirectly. Multiple functions of HMGB1 in DR are limited to murine models and in vitro studies. HIF-1α: hypoxia induced factor-1α; Egr-1: early growth response protein 1; TYK2: tyrosine kinase 2; CXCL12/CXCR4: chemokine; NOX2: nicotinamide adenine dinucleotide phosphate oxidase; RAGE: receptors for advanced glycation end-products; TLR-1/2/9/4: toll like receptor-1/2/9/4; ERK: extracellular signal-regulated kinase; NF-kB: nuclear factor-κB; ICAM-1: intercellular adhesion molecule-1; IL-1β/8: interleukin-1β/8; PLA-2: phospholipases A2; TNF-α: tumor necrosis factor-α; VEGF: vascular endothelial growth factor; SIRT1: sirtuin; PARP-1: poly ADP-ribose polymerase; ROS: reactive oxygen species; 8-OHdG: 8-hydroxydeoxyguanosine; VAP-1: vascular adhesion protein-1; HO-1: heme oxygenase-1; OPN: osteopontin.

At the moment, information regarding the function of HMGB1 in DR is mostly limited to murine models and in vitro studies. DM upregulates the expression of HMGB1, leading to the activation of inflammatory signaling pathways such as the RAGE-mediated activation of ERK1/2-NF-κB. Intravitreal injection of HMGB1 mimics the effects of diabetes and increases RAGE, ERK1/2, NF-κB, and proinflammatory biomarkers such as ICAM-1 and soluble ICAM-1 (Scheme 2). These mechanisms decrease TLR-2 and occludin expression, increasing retinal vascular permeability and disrupting the stability of tight junction complex between adjacent retinal microvascular EC [66].

High glucose stimulates the translocation of HMGB1 into the cytoplasm of retinal pericytes. RAGEs act as receptors for HMGB1 and, in diabetes, their expression is enhanced. HMGB1 is involved in the induction of DR through the activation of this receptor. HMGB1, through the binding of RAGEs, enhances the transcriptional activity of NF-κB in retinal pericytes in in vitro and in vivo models. Hyperglycemia also increases the binding of NF-κB to the RAGE promoter, inducing the overexpression of RAGEs and therefore establishing a vicious cycle [67].

HMGB1 is strictly related to the signal transducer and activator of transcription-3 (STAT-3). Constant intake of HMGB1 inhibitor glycyrrhizin (GA) attenuates the upregulation of phosphorylated STAT-3 (pSTAT-3). The inhibition of STAT-3 blocks HMGB1-induced VEGF upregulation and human retinal microvascular endothelial cell (HRMECs) migration, suggesting the role of STAT-3 in mediating HMGB1-induced angiogenesis in DR [68].

HMGB1 induces the significant upregulation of IL-1β and ROS and the expression of NOX2, caspase-3, and poly ADP-ribose polymerase-1 (PARP-1) in HRMECs [69].

HMGB1 may have a role in the alteration of BRB HMGB1 expression, which is enhanced in the retinas of diabetic rats, and BRB permeability is significantly increased [70].

Sirtuin 1 (SIRT1) is a member of the SIRT family of proteins with deacetylase activity. Many studies report its role in DNA repair, oxidative stress, angiogenesis, inflammation, and senescence. There is a strong link between SIRT1 expression and the development of DR and PDR. In particular, hyperglycemia and diabetes cause the downregulation of SIRT1, thus resulting in inflammation, angiogenesis, an increase in oxidative stress, and vascular permeability, all of which are hallmarks of diabetic damage [71]. There is a functional link between HMGB1 and SIRT1 in the regulation of the diabetes-induced breakdown of the BRB. Intravitreal injection of HMGB1 in normal rats results in the downregulation of SIRT1. The HMGB1 inhibitor GA attenuates the downregulation of and normalizes retinal SIRT1 expression. Moreover, treatment with the SIRT1 activator resveratrol attenuates the diabetes-induced downregulation of SIRT1, accompanied by reduced expression of HMGB1 and RAGEs. Resveratrol may confer protection against the diabetes-induced breakdown of BRB through SIRT1 upregulation and HMGB1 downregulation [72]. HMGB1, insulin-like growth factor-binding protein 3 (IGFBP-3), SIRT1, and protein kinase A (PKA) are strictly related. IGFBP-3 increases SIRT1 and decreases HMGB1. PKA mediates the reduction in cytoplasmic HMGB1 by increasing IGFBP-3 and SIRT1 activities [73].

Chen et al. found increased expression of HMGB1 and its receptor RAGEs TLR-2 and TLR-4 in the retinas of type 2 diabetic rats and human retinal pigment epithelial cell line-19 (ARPE-19) exposed to high glucose. The NF-κB activity was found to be increased as well. The blockage of HMGB1 downregulated NF-κB hyperactivation and VEGF production in high glucose cultured ARPE-19 cells [74].

High levels of HMGB1 expression are due to both gene transcription and protein synthesis. The specific mechanism by which HMGB1 leads to DR is unclear. It may exert its function via the TLR-9 pathway. The expression of TLR-9 was increased and positively related to the expression of HMGB1 [75].

A high glucose environment could promote HMGB1 expression and activate TLR-4 and NF-κB overexpression in retinal ganglion cells (RGC), thus leading to the inhibition of cell survival and growth. TLR-4 is an important receptor for HGMB-1 that is largely expressed in the nervous system and can regulate neuron growth and proliferation. When HMGB1 binds to TLR-4, it activates several signaling pathways such as NF-κB with the release of inflammatory cytokines, chemokines, and colony-stimulating factors, leading to leukocyte adhesion and inflammation [76]. Yu et al. showed a higher expression of HMGB1 in diabetic rats associated with the upregulation of phospholipases A2 (PLA-2), TNF-⍺, VEGF, and ICAM-1. Regarding HMGB1 receptors, RAGEs protein was increased, whereas TLR-1 was reduced, suggesting that HMGB1 effects are RAGE-mediated [77].

Injury and death of the retinal pericytes and EC in DR might be due to the HMGB1/PLA2 induced cytotoxic activity of glial cells as well as the direct effect of HMGB1 on EC. HMGB1 could mediate EC death directly and pericyte death indirectly through the HMGB1-induced cytotoxic activity of glial cells. Regarding PLA2 it seems to be a positive regulator of VEGF-induced angiogenesis [78].

HMGB1 has an important role in angiogenesis. It can act directly through RAGEs and TLR-4 with EC activation, proliferation, and migration. HMGB1 also promotes angiogenesis indirectly through the production of proangiogenic cytokines, such as VEGF, TNF-⍺, and IL-8 from EC and activated macrophages [79]. The same role of HMGB1 was also demonstrated by Santos et al. The authors suggest that HMGB1 is not able to mediate angiogenesis in the retina by itself [80].

According to Lee et al., AGEs cause a rise in intracellular ROS, inducing the release of HMGB1 into extracellular space. HMGB1 augments the signal via RAGEs or TLR and mediates the secretion of VEGF-A through the c-Jun N-terminal kinases signaling pathway that was blocked by HMGB1 inhibitor GA. This could be a possible way through which HMGB1 upregulates VEGF [81].

HMGB1 and VEGF-A expression are upregulated in serum samples of DR patients and are positively associated. The in vitro up-regulation of HMGB1 inhibits the retinal pigmented epithelium (RPE) cell viability and induces apoptosis. HMGB1 administration to RPE cells in high glucose conditions up-regulates the expression of VEGF-A [82].

The silencing of HMGB1 inhibits the activation of MAPK and NF-κB signaling pathway; modulates the levels of VEGF, ICAM-1 and vascular cell adhesion molecule-1 (VCAM-1), therefore influencing endothelial permeability; attenuates cell apoptosis, BRB damage, and the inflammatory response induced by high concentration of glucose [83].

HMGB1 may inhibit the expression of NF-κB light polypeptide gene enhancer in B-cell inhibitor-α, a protein capable of inhibiting NF-κB by binding to its promoter region. This determines the activation of the NF-κB pathway, influencing inflammation and angiogenic processes, thus leading to DR. High levels of HMGB1 stimulate apoptosis and inhibit the proliferation of human retinal endothelial cells (HRECs). HMGB1 may determine apoptosis through the NF-κB pathway thanks to an alternate mechanism of non-perfusion and neovascularization [84].

There is a potential link among HMGB1, vascular adhesion protein-1 (VAP-1), oxidative stress, and heme oxygenase-1 (HO-1) in the pathogenesis of inflammation and angiogenesis associated with PDR. HMGB1 levels are consistently increased in the vitreous of patients with PDR, particularly higher in patients with active PDR. Exogenous HMGB1 activates HRMECs to upregulate the adhesion molecule ICAM-1.

Increased levels of the oxidative marker 8-hydroxydeoxyguanosine (8-OHdG) in the vitreous of PDR patients, particularly in active PDR, have been found. The positive correlation between vitreous levels of HMGB1 and 8-OHdG in HRMECs suggests that HMGB1 is associated with oxidative stress.

Regarding VAP-1, there was a significant correlation between the levels of sVAP-1, HMGB1 concentration, and 8-OHdG in vitreous. Expression of VAP-1 was higher in diabetic patients compared to controls in the RPE, whereas no significant difference was found in the neuroretina.

Stimulation with HMGB1 caused the upregulation of HO-1 in HRMECs. HO-1 levels were significantly higher in eyes with active neovascularization compared with eyes with involuted PDR. These findings suggest that HO-1 might contribute to PDR angiogenesis and progression. Moreover, VEGF can induce the expression of HO-1 that stimulates the synthesis of VEGF in a positive feedback loop [85].

Vascular EC and stromal cells in diabetic epiretinal membranes express HMGB1, RAGE, osteopontin (OPN), and early growth response protein-1 (Egr-1). In diabetic epiretinal membranes, these proteins and receptors are specifically localized in myofibroblasts. This suggests that HMGB1/RAGE/OPN/Egr-1 signaling pathway is involved in the inflammatory, angiogenic, and fibrotic responses in proliferative vitreoretinopathy (PVR) and may contribute to the instauration of PDR and its most dangerous complications [86].

OPN, HMGB1, and connective tissue growth factor (CTGF) were upregulated in the vitreous of patients with PVR, particularly in their active form, whereas increased levels of pigment epithelium-derived factor (PEDF) may be a response designed to counteract the activity of the angiogenic and fibrogenic factors during the progression of PDR and PVR [87].

There is a relationship between the activity of PDR, the presence of vitreous hemorrhages, and levels of HMGB1. In fact, HMGB1 is higher in patients with active PDR compared with inactive PDR and is higher in PDR patients with vitreous hemorrhages compared with patients without it [88].

Shen et al. found that HMGB1, VEGF, RAGE, and IL-1β levels were significantly elevated in the vitreous and serum of patients with PDR, suggesting that the upregulation of HMGB1 might contribute to the initiation and progression of angiogenesis in PDR and that the HMGB1/RAGE signaling axis has a role in the progression of PDR [89].

The upregulation of HMGB1 can induce the downregulation of brain-derived neurotrophic factor (BDNF), a neurotrophin with a neurogenetic function, and also of synaptophysin, an integral membrane protein of synaptic vesicles involved in neurotransmission. HMGB1 upregulates cleaved caspase-3 in vitreous fluid and serum from patients with PDR, as well as in the retinas of diabetic rats. HMGB1 inhibitor GA is able to revert the downregulation of BDNF.

RAGEs and ICAMs levels are upregulated in the serum of patients with PDR. RAGEs bind its ligands, preventing their link to RAGE, therefore blocking the inflammatory cascade. Elevated levels of RAGEs in the serum of patients with PDR could negatively regulate inflammation and limit diabetes-induced retinal vascular and neuronal dysfunction [90].

HMGB1 and VEGF levels were higher in vitreous from PDR patients. Moreover, there were increased levels of soluble vascular endothelial-cadherin that could be a marker of EC activation or injury associated with angiogenesis, inflammation, and the breakdown of the inner BRB. Finally, there was lower angiogenic activity in patients with higher levels of soluble endoglin, suggesting that it could be protective against pathological angiogenesis [91].

The intravitreal injection of HMGB1 in normal rats mimics the effect of DM, with increased expression of HMGB1 protein and mRNA, caspase 3, and levels of glutamate (responsible for excitotoxic neuronal death). HMGB1 inhibitor glycyrrhizic acid attenuates all of these effects. The early retinal neuropathy induced by diabetes is, at least in part, attributable to the diabetes-induced upregulation of HMGB1. Inhibiting the release of HMGB1 with a constant intake of GA results in the reduction of diabetes-induced retinal neuropathy. This could be a novel therapeutic approach to DR. [92].

The induction of DM and intravitreal injection of HMGB1 in normal rats resulted in the significant upregulation of HIF-1α, Egr-1, tyrosine kinase 2 (TYK2), and the CXCL12/CXCR4 chemokine axis. HIF-1α is associated with retinal inflammation induced by diabetes, Egr-1 may play a role in the development of vascular complications of DM, and the CXCL12/CXCR4 chemokine axis contributes to neovascularization. All these upregulations are mediated by the interaction of HMGB1 with RAGE. Inhibition of the release of HMGB1, for example with GA, attenuates the upregulation of all these molecules [93].

Exposure to hypoxia is able to release HMGB1 from RPE cells. HMGB1 may stimulate the overproduction of angiogenic and fibrogenic factors such as VEGF and CTGF in RPE cells. HMGB1 is involved in DR pathogenesis through binding to TLR-4, RAGE, and their signaling cascades such as PI3K, p38/MAPK, and NF-κB [94,95].

## 4. Future Therapeutic Approaches

Numerous molecules have been studied as inhibitors of HMGB1 in recent years for the treatment and prevention of DR and its complications (Table 1).

### 4.1. Glycyrrhizin (GA)

GA is a triterpene glycoconjugate naturally extracted from licorice root (*Glycyrrhiza glabra*). It is composed of two molecules of glucuronic acid and glycyrrhetinic acid aglycone [96]. This molecule inhibits the chemotactic and pathogenic functions of HMGB1 by binding directly the A and B boxes [97]. GA shows a wide range of effects such as antibacterial, hepatoprotective, antiproliferative, antiallergic, and antiviral [98].

As a result of recent studies on ARPE cells, it has been demonstrated that HMGB1 is connected to the increase in angiogenesis and fibrosis during the course of DR [94]. Oral administration of GA in diabetic mice strongly inhibited HMGB1 concentration in retinas. This result led to a reduction in vascular and neuronal damage related to DR. The anti-inflammatory effects of GA were mediated by the inhibition of TNF-⍺, IL-1β, and the cleavage of caspase-3 in retinal EC. GA, through the inhibition of HMGB1, reduces ROS concentrations and blood circulating glucose [99]. In another work, GA reduced TLR-4 concentrations and ischemia-reperfusion damage as well as increasing the expression of insulin receptors, partially preserving the anatomical integrity of the retina [100]. In a recent study, Liu et al. demonstrated that exchange protein for cAMP1, an inflammatory molecule involved in leukostasis, acts in synergy with GA. The combination of these two proteins strongly inhibits HMGB1 through the activation of SIRT1. SIRT1 deacetylates HMGB1, exerting a protective role in the diabetic retina [101]. GA also suppresses the proangiogenic effects of HMGB1 as it blocks AGE-induced upregulation of VEGF [81]. Abu El-Asrar and Mohammad’s workgroup demonstrated, in a diabetic murine model, that GA can inhibit HMGB1’s cytokine-like activities. Specifically, oral GA determines a reduction in HIF-1α, transcription factor Egr-1, TYK2, CXCL12, and CXCR4 [93]. Moreover, the same authors demonstrated that GA can inhibit the upregulation of STAT-3 induced by HMGB1 and its translocation in retinal Müller cells [68], upregulate BDNF expression in experimental mice [90], attenuate the expression of NOX2, caspase-3, and PARP-1 in the retinas as well as lowering the concentrations of ROS [69] and cleave caspase-3 glutamate and downregulating neurodegeneration mediators and markers in murine retinas [92] and attenuating the expression of retinal ICAM-1 [84]; lastly, it inhibits HMGB1 mediated activation of NF-κB. [66]. GA in association with resveratrol shows the ability to replenish retinal SIRT1 expression [72]. 

All this evidence points in the direction of the potential use of GA in the prevention of DR and its complications. It is worth mentioning that, in human studies on male patients affected by chronic hepatitis and type 2 diabetes, the administration of GA reduced serum testosterone aggravating insulin resistance, atherosclerosis, and sexual dysfunctions [102]. Further studies, especially on human subjects, are needed in order to confirm the pathways and molecules involved and their efficacy and safety.

### 4.2. Small Interfering RNAs/Short Hairpin RNA (siRNA/shRNA) 

Small interfering RNAs (siRNAs) are a class of double-strand RNA usually constituted by 21–25 nucleotides that are gaining importance as therapeutic tools in numerous diseases. SiRNAs are capable of selectively binding specific genomic sequences, silencing them and therefore inhibiting the protein expression [103,104].

SiRNA HMGB1 transfection can repress HMGB1 RNA overexpression, determining the suppression of TLR-4 and NF-κB mRNA in RGCs. The downregulation of these inflammatory pathways can promote the survival and growth rates of RGCs [76]. A similar study conducted by Jiang and Chen confirmed these results in both in vivo and in vitro models. HMGB1 suppression, mediated by intravitreal injections of siRNA, is capable, in diabetic rats, of reducing retinal apoptosis rates as well as improving retinal function. In HRECs exposed to high glucose concentrations, siRNA HMGB1 improved cell viability and reduced the oxidative damage lowering ROS production [105]. The same study group demonstrated the protective role of HMGB1 inhibition in murine DR models. Retinal cells isolated from 8-year-old rats were incubated with a recombinant lentivirus containing short hairpin RNA (shRNA) for HMGB1. Through this mechanism, the authors obtained the silencing of HMGB1 gene expression. The results showed the downregulation of both MAPK and NF-κB, contributing to the reduction of inflammation, cell death, and BRB breakdown [83].

### 4.3. Polygonum Cuspidatum (PCE)

*P. cuspidatum*, also known as “Hojang-geun” in Korea, is a commonly employed herbal medicine in East Asia. The plant shows anti-inflammatory and anti-diabetic effects [106]. Recent works have explored the potential role, as a preventive treatment, of *P. cuspidatum* extract in diabetic nephropathy [107]. PCE is rich in resveratrol, polidatyn, and emodin compounds, with strong anti-inflammatory properties [108]. Sohn et al. suggested a beneficial effect of the ethanol extract of the root in a DR murine model. It prevents diabetic-induced retinal vascular hyperpermeability, attenuating the HMGB1 signaling pathway through the downregulation of the RAGE-mediated activation of NF-κB. It directly blocks the binding of HMGB1 to RAGE, thus preventing retinal vascular inflammation. Moreover, fluorescein angiography demonstrated that PCE markedly inhibits fluorescein leakage, suggesting that it may prevent the breakdown of the BRB. PCE reduces the expression of HMGB1 in diabetic rat retinal tissue and inhibits the binding of NF-κB to the RAGE promoter, with considerable anti-inflammatory activity. It is worth mentioning that the oral administration of PCE showed no positive effects on glycemic and body weight control in the murine model [109].

### 4.4. Paeoniflorin

Paeoniflorin is a monoterpene glucoside extracted from the root of the *Paeonia Lactiflora*. Paeoniflorin shows anti-inflammatory properties and it is already used in traditional Chinese medicine for a wide range of pathologies [110,111]. Moreover, paeoniflorin shows immunomodulatory effects on microglial cells through the enhancement of the suppressor of cytokine signaling 3 (SOCS3) pathways [112]. Zhu et al. demonstrated, in an in vitro study on BV2 microglial cells exposed to high concentrations of glucose, that treatment with paeoniflorin reduces the expression of metalloproteinases-9 (MMP-9) through the inhibition of p38/NF-κB. In addition, paeoniflorin activates SOCS3, which blocks the TLR-4 pathway [112,113]. The repression of TLR-4 determines the reduction of HMGB1 mediated inflammation in retinal microglial cells [114].

### 4.5. Salicin

Salicin is the main component of the willow bark extract, commonly used in traditional medicine for its anti-inflammatory and antipyretic effects [115]. Salicin is metabolized to salicylic acid in vivo; therefore, it is also known as “nature’s aspirin”. Previous studies showed that salicin exerts protective effects on EC, both inhibiting angiogenesis [116] and reducing ROS production [117]. Song et al. demonstrated that the treatment of HRECs with salicin led to a reduction in HMGB1 release and the prevention of cellular apoptosis. Moreover, the authors demonstrated that salicin can suppress the production of IL-1β and its related cytokines, such as TNF-⍺ and IL-6, responsible for retinal toxicity. Salicin is also able to block the release of the adhesion molecules ICAM-1 and VCAM-1 and the NF-κB inflammatory pathway [118].

### 4.6. Ethyl Pyruvate (EP)

Ethyl pyruvate is a pyruvate derivative with the addition of an aliphatic ester group. EP is considered to be safer and more effective than pyruvate in inhibiting ROS and inflammation [119]. EP is a strong HMGB1 inhibitor. Treatment with EP promotes stable vascular growth and blocks retinal pathological neovascularization by preventing the overexpression of HMGB1. Moreover, EP can inhibit the expression of IL-6, TNF-⍺, and NF-kB, exerting a protective role in chronic inflammatory diseases such as DR [120].

### 4.7. Bradykinin (BK)

BK is a vasoactive peptide part of the kinins family. BK participates in several processes such as inflammation, pain, and cell proliferation [121]. Zhu et al. studied the effects of BK in HRECs exposed to high concentrations of glucose. Results show that BK can suppress oxidative stress and the release of inflammatory mediators. It can also control the process of neovascularization, downregulating the expression of VEGF. Lastly, BK inhibits the HMGB1/NF-κB signaling pathway, therefore controlling the growth and proliferation of HRECs [122].

### 4.8. Kallistatin

Kallistatin is an endogenous serine proteinase that plays numerous physiological and pathological roles like tumorigenesis, vasodilation, inhibition of neovascularization, inflammation, oxidative stress, cellular death, and fibrosis [123]. It stimulates the expression of eNOS, SIRT1, and SOCS3, while it inhibits VEGF, HMGB1, TNF-⍺, and NF-κB [124]. It has been demonstrated that vitreous humor levels of kallistatin in patients with PDR are lower when compared to healthy controls [125]. Xing et al. established that kallistatin is a strong inhibitor of angiogenesis and therefore may act as a potent drug in the prevention of PDR [126].

### 4.9. Compound 49b

Compound 49b is a recently discovered β-adrenergic receptor agonist that has already demonstrated efficacy in preventing apoptosis in in vitro models of EC and Müller cells exposed to high glucose [127,128]. Recent evidence shows that compound 49b can inhibit HMGB1 expression, TLR-4 downstream signaling, and, therefore, NF-κB in both EC and Müller cells. This leads to the idea that this agonist may preserve vascular and neuronal integrity in the diabetic retina [126,129].

### 4.10. Cyclosporine A (CyA)

Cyclosporine A is a polypeptide derived from the fungi *Beauveria nevus* and *Tolypocladium inflatum*, and it is well known for its anti-inflammatory and immunosuppressive effects [130]. Wang et al. demonstrated that CyA attenuates the enhanced expression of IL-1β and TNF-⍺ in the retinas of diabetic rats, probably via the suppression of HMGB1. The intravitreal injection of CyA may represent a novel therapeutic strategy to treat DR [131].

CyA is also involved in the reduction of BRB permeability in diabetic rats. In particular, it reduces the levels of IL-1β, nitric oxide (through a decreased expression of iNOS), and IL-1β-induced cyclooxygenase-2 (COX-2) expression. Moreover, CyA decreases vitreous protein concentration in diabetic rats. The authors suggest that this reduction in vitreous protein concentration can be linked to the reduction of BRB permeability [132].

## 5. Conclusions

The purpose of this review is to show the role of HMGB1 in DR. Many studies have demonstrated that DM, and then hyperglycemia, upregulate the expression of and increase in the levels of HMGB1. This situation activates several pathways and involves a large number of molecules, such as ERK, NF-κB, ICAM, RAGE, VEGF, and TLR. The final result is the activation and increase of inflammation, angiogenesis, oxidative stress, and, ultimately, retinal damage to the patients. At the same time, the involvement of this great number of molecules can provide a hint of new therapeutic approaches to be developed and studied.

## Figures and Tables

**Table 1:** Summary of direct and indirect high-mobility group box (HMGB1) inhibitors with therapeutic potential in diabetic retinopathy. HRECs = human retinal endothelial cells; TLR = toll like receptor; TNF-α = tumor necrosis factor-α; GCL = ganglion cell layer; STZ = streptozotocin; SIRT1 = sirtuin 1; BDNF = brain-derived neurotrophic factor; ROS = reactive oxygen species; ICAM-1= intercellular adhesion molecule-1; NF-κB = nuclear factor-κB; HIF-1α = hypoxia induced factor-1α; pSTAT-3 = phosphorylated signal transducer and activator of transcription-3; VEGF = vascular endothelial growth factor; siRNA = small interfering RNAs; shRNA = short hairpin RNA; RAGE = receptors for AGEs; MMP = metalloproteinases; IL = interleukin; SOCS3 = suppressor of cytokine signaling 3; ROP = Retinopathy of prematurity; SOD = superoxide dismutase; IGFBP-3 = insulin-like growth factor-binding protein-3; iNOS = inducible nitric oxide synthase; COX-2 = cyclooxygenase; BRB = blood retinal barrier.

Drug	Target Test	Diabetic Inducement	Mechanism of Action	Results	Reference
***Glycyrrhizin***	HRECs	High glucose concentrations	Inhibition of TLR-4 and TNF-α; cleavage of caspase 3 through inactivation of HMGB1	Increased insulin receptor signal transduction	[99,100]
	Mice	Ischemia/reperfusion damage	Block of the loss of retinal thickness	Protects GCL and retinal capillaries	[99,100]
	Mice	STZ	Upregulation of SIRT1, inhibition of inflammatory factors. Attenuates BDNF downregulation, reduces ROS, ICAM-1, NF-κB, and HIF-1α	Reduced vascular permeability, increased retinal thickness. Protection from diabetes-induced retinal damages and inflammation	[101] [67,68,85,90] [93]
	Retinal Muller Cells	High glucose concentrations	Attenuates p-STAT3 expression	Inhibition of VEGF expression	[69]
***Small interfering RNAs***	Mice	STZ	Intravitreal injection of HMGB1 siRNA	Protected morphological changes, and improved the function of the retina	[105]
	Retinal ganglion cells	High glucose concentrations	Transfection with HMGB1 siRNA reduced the expression of TLR-4 and NF-κB	Increased cell survival rate	[77]
	HRECs	High glucose concentrations	Transfection with HMGB1 siRNA reduced the expression of caspase 3	Inhibition the early stage of apoptosis	[105]
***Short hairpin RNAs***	Rat retinas	High glucose concentrations	Transfection with HMGB1 shRNA reduced the expression TNF-α and NF-κB	Increased cell survival rate and vascular permeability	[83]
***Polygonum cuspidatum***	Mice	STZ	Reduced RAGE and NF-κB expression	Reduced vascular permeability	[109]
***Paeoniflorin***	Mice	STZ	Inhibition of MMP-9 and IL-1β	Alleviated microglial activation	[113]
	BV2 modified microglial cells	High glucose concentrations	Inhibition of NF-κB expression and SOCS3	Reduced MMP-9 and TLR-4 concentrations	[113]
***Salicin***	HRECs	Incubated with IL-1β (inflammatory response)	Suppression of NF-κB pathway and the release of MMP	Inhibition of IL-1β mediated inflammatory pathways	[118]
***Ethyl pyruvate***	Mice	Induction of ROP through exposition to hyperoxia	Reduction of ROS, NF-κB, IL-6, VEGF and TNF-α	Reduction of neoangiogenesis and areas of ischemic retina	[120]
***Bradykinin***	HRECs	High glucose concentrations	Suppression of NF-κB, caspase 3, VEGF, TNF-α, IL-1β. Increase in SOD activity	Promotion of retinal cells survival/ inhibition of apoptosis. Reduction of vascular permeability	[122]
***Kallistatin***	HRECs	High glucose concentrations	Suppression of VEGF expression	Reduction of neoangiogenesis	[126]
***Compound 49b***	HRECs and rat retinal Muller cells	High glucose concentrations	Increase of IGFBP-3 levels and inhibition of TLR-4 pathway	Prevention of cellular apoptosis	[127,129]
	Mice	SZT	Increase of IGFBP-3 levels	Prevention of the decrease in retinal thickness and loss of cells in GCL	[127]
***Cyclosporine A***	Mice	STZ	Reduction of TNF-α and IL-1β	Amelioration of retinal thickness, regression of retinal edema	[131]
	Mice	STZ	Reduction of iNOS, IL-1β and COX-2	Reduction of BRB permeability	[132]

## Abbreviations

8-OHdG	8-hydroxydeoxyguanosine
AGE	advanced glycation end-products
ARPE-19	adult retinal pigment epithelial cell line-19
BDNF	brain-derived neurotrophic factor
BK	bradykinin
BRB	blood–retinal barrier
COX-2	cyclooxygenase
CTGF	connective tissue growth factor
CyA	cyclosporine A
DM	diabetes mellitus
DR	diabetic retinopathy
EC	endothelial cells
Egr-1	early growth response protein 1
eNOS	endothelial nitric oxide synthase
EP	ethyl pyruvate
ERK	extracellular signal-regulated kinase
GA	glycyrrhizin
GSH	reduced glutathione
HIF-1α	hypoxia induced factor-1α
HMGB	high-mobility group box
HO-1	heme oxygenase-1
HRECs	human retinal endothelial cells
HRMEC	human retinal microvascular endothelial cells
ICAM-1	intercellular adhesion molecule-1
IFN	interferon
IGFBP-3	insulin-like growth factor-binding protein-3
IL	interleukin
iNOS	inducible nitric oxide synthase
MAPK	mitogen-activated protein kinase
MMP-9	metalloproteinases-9
NF-κB	nuclear factor-κB
NOX	nicotinamide adenine dinucleotide phosphate oxidase
NPDR	non proliferative diabetic retinopathy
OPN	osteopontin
PAI-1	plasminogen activator inhibitor-1
PARP	poly ADP-ribose polymerase
PCE	polygonum cuspidatum
PDR	proliferative diabetic retinopathy
PEDF	pigment epithelium-derived factor
PI3K	phosphatidylinositol 3-kinase
PK	protein kinase
PLA-2	phospholipases A2
PVR	proliferative vitreoretinopathy
RAGE	receptors for AGEs
RGC	retinal ganglion cells
ROP	retinopathy of prematurity
ROS	reactive oxygen species
RPE	retinal pigmented epithelium
shRNA	short hairpin RNA
siRNA	small interfering RNAs
SIRT	sirtuin
SOCS3	suppressor of cytokine signaling 3
SOD	superoxide dismutase
STAT-3	signal transducer and activator of transcription-3
pSTAT-3	phosphorylated STAT-3
STZ	streptozotocin
TGF-β1	transforming growth factor-β1
TLR	toll like receptor
TNF-α	tumor necrosis factor-α
TYK2	tyrosine kinase 2
VAP-1	vascular adhesion protein-1
VCAM-1	vascular cell adhesion molecule-1
VEGF	vascular endothelial growth factor

## References

[1] International Diabetes Federation (2019). IDF Diabetes Atlas.

[2] Yau J.W., Rogers S.L., Kawasaki R., Lamoureux E.L., Kowalski J.W., Bek T., Chen S.J., Dekker J.M., Fletcher A., Grauslund J. (2012). Global prevalence and major risk factors of diabetic retinopathy. Diabetes Care.

[3] Flaxman S.R., Bourne R.R.A., Resnikoff S., Ackland P., Braithwaite T., Cicinelli M.V., Das A., Jonas J.B., Keeffe J., Kempen J.H. (2017). Global causes of blindness and distance vision impairment 1990–2020: A systematic review and meta-analysis. Lancet Glob. Health.

[4] Cheung N., Mitchell P., Wong T.Y. (2010). Diabetic retinopathy. Lancet.

[5] Hendrick A.M., Gibson M.V., Kulshreshtha A. (2015). Diabetic Retinopathy. Prim. Care.

[6] Robles-Rivera R.R., Castellanos-González J.A., Olvera-Montaño C., Flores-Martin R.A., López-Contreras A.K., Arevalo-Simental D.E., Cardona-Muñoz E.G., Roman-Pintos L.M., Rodríguez-Carrizalez A.D. (2020). Adjuvant Therapies in Diabetic Retinopathy as an Early Approach to Delay Its Progression: The Importance of Oxidative Stress and Inflammation. Oxid. Med. Cell. Longev..

[7] Sohn E.H., van Dijk H.W., Jiao C., Kok P.H., Jeong W., Demirkaya N., Garmager A., Wit F., Kucukevcilioglu M., van Velthoven M.E. (2016). Retinal neurodegeneration may precede microvascular changes characteristic of diabetic retinopathy in diabetes mellitus. Proc. Natl. Acad. Sci. USA.

[8] Bandello F., Lattanzio R., Zucchiatti I., Del Turco C. (2013). Pathophysiology and treatment of diabetic retinopathy. Acta Diabetol..

[9] Semeraro F., Morescalchi F., Cancarini A., Russo A., Rezzola S., Costagliola C. (2019). Diabetic retinopathy, a vascular and inflammatory disease: Therapeutic implications. Diabetes Metab..

[10] Stitt A.W. (2010). AGEs and Diabetic Retinopathy. Investig. Ophthalmol. Vis. Sci..

[11] Testa R., Bonfigli A.R., Prattichizzo F., La Sala L., De Nigris V., Ceriello A. (2017). The “Metabolic Memory” Theory and the Early Treatment of Hyperglycemia in Prevention of Diabetic Complications. Nutrients.

[12] Rodríguez M.L., Pérez S., Mena-Mollá S., Desco M.C., Ortega Á.L. (2019). Oxidative Stress and Microvascular Alterations in Diabetic Retinopathy: Future Therapies. Oxid. Med. Cell. Longev..

[13] Kowluru R.A., Abbas S.N. (2003). Diabetes-induced mitochondrial dysfunction in the retina. Investig. Ophthalmol. Vis. Sci..

[14] Chalupsky K., Cai H. (2005). Endothelial dihydrofolate reductase: Critical for nitric oxide bioavailability and role in angiotensin II uncoupling of endothelial nitric oxide synthase. Proc. Natl. Acad. Sci. USA.

[15] Geraldes P., Hiraoka-Yamamoto J., Matsumoto M., Clermont A., Leitges M., Marette A., Aiello L.P., Kern T.S., King G.L. (2009). Activation of PKC-δ and SHP-1 by hyperglycemia causes vascular cell apoptosis and diabetic retinopathy. Nat. Med..

[16] Brownlee M. (2005). The pathobiology of diabetic complications: A unifying mechanism. Diabetes.

[17] Kowluru R.A., Mishra M. (2015). Oxidative stress, mitochondrial damage and diabetic retinopathy. Biochim. Biophys. Acta.

[18] Hammes H.P. (2018). Diabetic retinopathy: Hyperglycemia, oxidative stress and beyond. Diabetologia.

[19] Yao D., Taguchi T., Matsumura T., Pestell R., Edelstein D., Giardino I., Suske G., Rabbani N., Thornalley P.J., Sarthy V.P. (2007). High glucose increases angiopoietin-2 transcription in microvascular endothelial cells through methylglyoxal modification of mSin3A. J. Biol. Chem..

[20] Rajamani U., Jialal I. (2014). Hyperglycemia induces Toll-like receptor-2 and -4 expression and activity in human microvascular retinal endothelial cells: Implications for diabetic retinopathy. J. Diabetes Res..

[21] Wang Y.L., Wang K., Yu S.J., Li Q., Li N., Lin P.Y., Li M.M., Guo J.Y. (2015). Association of the TLR4 signaling pathway in the retina of streptozotocin-induced diabetic rats. Graefes Arch. Clin. Exp. Ophthalmol..

[22] PKC-DRS Study Group (2005). The effect of ruboxistaurin on visual loss in patients with moderately severe to very severe nonproliferative diabetic retinopathy: Initial results of the Protein Kinase C beta Inhibitor Diabetic Retinopathy Study (PKC-DRS) multicenter randomized clinical trial. Diabetes.

[23] Wang W., Lo A.C.Y. (2018). Diabetic Retinopathy: Pathophysiology and Treatments. Int. J. Mol. Sci..

[24] Lee R., Wong T.Y., Sabanayagam C. (2015). Epidemiology of diabetic retinopathy, diabetic macular edema and related vision loss. Eye Vis. (Lond.).

[25] Goodwin G.H., Sanders C., Johns E.W. (1973). A new group of chromatin-associated proteins with a high content of acidic and basic amino acids. Eur. J. Biochem..

[26] Tripathi A., Shrinet K., Kumar A. (2019). HMGB1 protein as a novel target for cancer. Toxicol. Rep..

[27] Kang R., Chen R., Zhang Q., Hou W., Wu S., Cao L., Huang J., Yu Y., Fan X.G., Yan Z. (2014). HMGB1 in health and disease. Mol. Aspects Med..

[28] Yang H., Antoine D.J., Andersson U., Tracey K.J. (2013). The many faces of HMGB1: Molecular structure-functional activity in inflammation, apoptosis, and chemotaxis. J. Leukoc. Biol..

[29] Stumbo A.C., Cortez E., Rodrigues C.A., Henriques M.D., Porto L.C., Barbosa H.S., Carvalho L. (2008). Mitochondrial localization of non-histone protein HMGB1 during human endothelial cell-Toxoplasma gondii infection. Cell Biol. Int..

[30] Tang D., Kang R., Zeh H.J., Lotze M.T. (2011). High-mobility group box 1, oxidative stress, and disease. Antioxid. Redox Signal..

[31] Venereau E., Casalgrandi M., Schiraldi M., Antoine D.J., Cattaneo A., De Marchis F., Liu J., Antonelli A., Preti A., Raeli L. (2012). Mutually exclusive redox forms of HMGB1 promote cell recruitment or proinflammatory cytokine release. J. Exp. Med..

[32] Kazama H., Ricci J.E., Herndon J.M., Hoppe G., Green D.R., Ferguson T.A. (2008). Induction of immunological tolerance by apoptotic cells requires caspase-dependent oxidation of high-mobility group box-1 protein. Immunity.

[33] Mandke P., Vasquez K.M. (2019). Interactions of high mobility group box protein 1 (HMGB1) with nucleic acids: Implications in DNA repair and immune responses. DNA Repair (Amst.).

[34] Tang D., Kang R., Livesey K.M., Cheh C.W., Farkas A., Loughran P., Hoppe G., Bianchi M.E., Tracey K.J., Zeh H.J. (2010). Endogenous HMGB1 regulates autophagy. J. Cell Biol..

[35] Tadie J.M., Bae H.B., Jiang S., Park D.W., Bell C.P., Yang H., Pittet J.F., Tracey K., Thannickal V.J., Abraham E. (2013). HMGB1 promotes neutrophil extracellular trap formation through interactions with Toll-like receptor 4. Am. J. Physiol. Lung Cell. Mol. Physiol..

[36] Ciucci A., Gabriele I., Percario Z.A., Affabris E., Colizzi V., Mancino G. (2011). HMGB1 and cord blood: Its role as immuno-adjuvant factor in innate immunity. PLoS ONE.

[37] Li R., Wang J., Zhu F., Li R., Liu B., Xu W., He G., Cao H., Wang Y., Yang J. (2018). HMGB1 regulates T helper 2 and T helper17 cell differentiation both directly and indirectly in asthmatic mice. Mol. Immunol..

[38] Lee G., Espirito Santo A.I., Zwingenberger S., Cai L., Vogl T., Feldmann M., Horwood N.J., Chan J.K., Nanchahal J. (2018). Fully reduced HMGB1 accelerates the regeneration of multiple tissues by transitioning stem cells to GAlert. Proc. Natl. Acad. Sci. USA.

[39] Andersson U., Erlandsson-Harris H., Yang H., Tracey K.J. (2002). HMGB1 as a DNA-binding cytokine. J. Leukoc. Biol..

[40] Park J.S., Arcaroli J., Yum H.K., Yang H., Wang H., Yang K.Y., Choe K.H., Strassheim D., Pitts T.M., Tracey K.J. (2003). Activation of gene expression in human neutrophils by high mobility group box 1 protein. Am. J. Physiol. Cell. Physiol..

[41] He Q., You H., Li X.M., Liu T.H., Wang P., Wang B.E. (2012). HMGB1 promotes the synthesis of pro-IL-1β and pro-IL-18 by activation of p38 MAPK and NF-κB through receptors for advanced glycation end-products in macrophages. Asian Pac. J. Cancer Prev..

[42] Wu X., Mi Y., Yang H., Hu A., Zhang Q., Shang C. (2013). The activation of HMGB1 as a progression factor on inflammation response in normal human bronchial epithelial cells through RAGE/JNK/NF-κB pathway. Mol. Cell. Biochem..

[43] Andersson U., Wang H., Palmblad K., Aveberger A.C., Bloom O., Erlandsson-Harris H., Janson A., Kokkola R., Zhang M., Yang H. (2000). High mobility group 1 protein (HMG-1) stimulates proinflammatory cytokine synthesis in human monocytes. J. Exp. Med..

[44] Sundberg E., Fasth A.E., Palmblad K., Harris H.E., Andersson U. (2009). High mobility group box chromosomal protein 1 acts as a proliferation signal for activated T lymphocytes. Immunobiology.

[45] Degryse B., de Virgilio M. (2003). The nuclear protein HMGB1, a new kind of chemokine?. FEBS Lett..

[46] Schlueter C., Weber H., Meyer B., Rogalla P., Röser K., Hauke S., Bullerdiek J. (2005). Angiogenetic signaling through hypoxia: HMGB1: An angiogenetic switch molecule. Am. J. Pathol..

[47] Yang S., Xu L., Yang T., Wang F. (2014). High-mobility group box-1 and its role in angiogenesis. J. Leukoc. Biol..

[48] Harris H.E., Andersson U., Pisetsky D.S. (2012). HMGB1: A multifunctional alarmin driving autoimmune and inflammatory disease. Nat. Rev. Rheumatol..

[49] Yang H., Wang H., Ju Z., Ragab A.A., Lundbäck P., Long W., Valdes-Ferrer S.I., He M., Pribis J.P., Li J. (2015). MD-2 is required for disulfide HMGB1-dependent TLR4 signaling. J. Exp. Med..

[50] Yang H., Wang H., Andersson U. (2020). Targeting Inflammation Driven by HMGB1. Front. Immunol..

[51] Scaffidi P., Misteli T., Bianchi M.E. (2002). Release of chromatin protein HMGB1 by necrotic cellstriggers inflammation. Nature.

[52] Hofmann M.A., Drury S., Fu C., Qu W., Taguchi A., Lu Y., Avila C., Kambham N., Bierhaus A., Nawroth P. (1999). RAGE mediates a novel proinflammatory axis: A central cell surface receptor for S100/calgranulin polypeptides. Cell.

[53] Toure F., Zahm J.M., Garnotel R., Lambert E., Bonnet N., Schmidt A.M., Vitry F., Chanard J., Gillery P., Rieu P. (2008). Receptor for advanced glycationend-products (RAGE) modulates neutrophil adhesion and migration on glycoxidated extracellular matrix. Biochem. J..

[54] Sims G.P., Rowe D.C., Rietdijk S.T., Herbst R., Coyle A.J. (2010). HMGB1 and RAGE in inflammation and cancer. Annu. Rev. Immunol..

[55] Zhang C., Dong H., Chen F., Wang Y., Ma J., Wang G. (2019). The HMGB1-RAGE/TLR-TNF-α signaling pathway may contribute to kidney injury induced by hypoxia. Exp. Ther. Med..

[56] Massey N., Puttachary S., Bhat S.M., Kanthasamy A.G., Charavaryamath C. (2019). HMGB1-RAGE Signaling Plays a Role in Organic Dust-Induced Microglial Activation and Neuroinflammation. Toxicol. Sci..

[57] Wolfson R.K., Chiang E.T., Garcia J.G. (2011). HMGB1 induces human lung endothelial cell cytoskeletal rearrangement and barrier disruption. Microvasc. Res..

[58] Bangert A., Andrassy M., Müller A.M., Bockstahler M., Fischer A., Volz C.H., Leib C., Göser S., Korkmaz-Icöz S., Zittrich S. (2016). Critical role of RAGE and HMGB1 in inflammatory heart disease. Proc. Natl. Acad. Sci. USA.

[59] Tang D., Shi Y., Kang R., Li T., Xiao W., Wang H., Xiao X. (2007). Hydrogen peroxide stimulates macrophages and monocytes to actively release HMGB1. J. Leukoc. Biol..

[60] Mitola S., Belleri M., Urbinati C., Coltrini D., Sparatore B., Pedrazzi M., Melloni E., Presta M. (2006). Cutting edge: Extracellular high mobility group box-1 protein is a proangiogenic cytokine. J. Immunol..

[61] van Beijnum J.R., Nowak-Sliwinska P., van den Boezem E., Hautvast P., Buurman W.A., Griffioen A.W. (2013). Tumor angiogenesis is enforced by autocrine regulation of high-mobility group box 1. Oncogene.

[62] Wu C.Z., Zheng J.J., Bai Y.H., Xia P., Zhang H.C., Guo Y. (2018). HMGB1/RAGE axis mediates the apoptosis, invasion, autophagy, and angiogenesis of the renal cell carcinoma. OncoTargets Ther..

[63] Lin Q., Yang X.P., Fang D., Ren X., Zhou H., Fang J., Liu X., Zhou S., Wen F., Yao X. (2011). High-mobility group box-1 mediates toll-like receptor 4-dependent angiogenesis. Arterioscler. Thromb. Vasc. Biol..

[64] Jiang W., Jiang P., Yang R., Liu D.F. (2018). Functional role of SIRT1-induced HMGB1 expression and acetylation in migration, invasion and angiogenesis of ovarian cancer. Eur. Rev. Med. Pharmacol. Sci..

[65] Park S.Y., Lee S.W., Kim H.Y., Lee W.S., Hong K.W., Kim C.D. (2015). HMGB1 induces angiogenesis in rheumatoid arthritis via HIF-1α activation. Eur. J. Immunol..

[66] Mohammad G., Siddiquei M.M., Othman A., Al-Shabrawey M., Abu El-Asrar A.M. (2013). High-mobility group box-1 protein activates inflammatory signaling pathway components and disrupts retinal vascular-barrier in the diabetic retina. Exp. Eye Res..

[67] Kim J., Kim C.S., Sohn E., Kim J.S. (2016). Cytoplasmic translocation of high-mobility group box-1 protein is induced by diabetes and high glucose in retinal pericytes. Mol. Med. Rep..

[68] Mohammad G., Jomar D., Siddiquei M.M., Alam K., Abu El-Asrar A.M. (2017). High-Mobility Group Box-1 Protein Mediates the Regulation of Signal Transducer and Activator of Transcription-3 in the Diabetic Retina and in Human Retinal Müller Cells. Ophthalmic Res..

[69] Mohammad G., Alam K., Nawaz M.I., Siddiquei M.M., Mousa A., Abu El-Asrar A.M. (2015). Mutual enhancement between high-mobility group box-1 and NADPH oxidase-derived reactive oxygen species mediates diabetes-induced upregulation of retinal apoptotic markers. J. Physiol. Biochem..

[70] Ran R.J., Zheng X.Y., Du L.P., Zhang X.D., Chen X.L., Zhu S.Y. (2016). Upregulated inflammatory associated factors and blood-retinal barrier changes in the retina of type 2 diabetes mellitus model. Int. J. Ophthalmol..

[71] Zhou M., Luo J., Zhang H. (2018). Role of Sirtuin 1 in the pathogenesis of ocular disease (Review). Int. J. Mol. Med..

[72] Mohammad G., Abdelaziz G.M., Siddiquei M.M., Ahmad A., De Hertogh G., Abu El-Asrar A.M. (2019). Cross-Talk between Sirtuin 1 and the Proinflammatory Mediator High-Mobility Group Box-1 in the Regulation of Blood-Retinal Barrier Breakdown in Diabetic Retinopathy. Curr. Eye Res..

[73] Liu L., Patel P., Steinle J.J. (2018). PKA regulates HMGB1 through activation of IGFBP-3 and SIRT1 in human retinal endothelial cells cultured in high glucose. Inflamm. Res..

[74] Chen X.L., Zhang X.D., Li Y.Y., Chen X.M., Tang D.R., Ran R.J. (2013). Involvement of HMGB1 mediated signalling pathway in diabetic retinopathy: Evidence from type 2 diabetic rats and ARPE-19 cells under diabetic condition. Br. J. Ophthalmol..

[75] Jiang S., Chen X. (2017). Expression of High-Mobility Group Box 1 Protein (HMGB1) and Toll-Like Receptor 9 (TLR9) in Retinas of Diabetic Rats. Med. Sci. Monit..

[76] Zhao H., Zhang J., Yu J. (2015). HMGB-1 as a Potential Target for the Treatment of Diabetic Retinopathy. Med. Sci. Monit..

[77] Yu Y., Yang L., Lv J., Huang X., Yi J., Pei C., Shao Y. (2015). The role of high mobility group box 1 (HMGB-1) in the diabetic retinopathy inflammation and apoptosis. Int. J. Clin. Exp. Pathol..

[78] Gong Y., Jin X., Wang Q.S., Wei S.H., Hou B.K., Li H.Y., Zhang M.N., Li Z.H. (2014). The involvement of high mobility group 1 cytokine and phospholipases A2 in diabetic retinopathy. Lipids Health Dis..

[79] van Beijnum J.R., Buurman W.A., Griffioen A.W. (2008). Convergence and amplification of toll-like receptor (TLR) and receptor for advanced glycation end products (RAGE) signaling pathways via high mobility group B1 (HMGB1). Angiogenesis..

[80] Santos A.R., Dvoriantchikova G., Li Y., Mohammad G., Abu El-Asrar A.M., Wen R., Ivanov D. (2014). Cellular mechanisms of high mobility group 1 (HMGB-1) protein action in the diabetic retinopathy. PLoS ONE.

[81] Lee J.J., Hsiao C.C., Yang I.H., Chou M.H., Wu C.L., Wei Y.C., Chen C.H., Chuang J.H. (2012). High-mobility group box 1 protein is implicated in advanced glycation end products-induced vascular endothelial growth factor A production in the rat retinal ganglion cell line RGC-5. Mol. Vis..

[82] Fu D., Tian X. (2015). Effect of high mobility group box 1 on the human retinal pigment epithelial cell in high-glucose condition. Int. J. Clin. Exp. Med..

[83] Jiang N., Chen X. (2017). Protective effect of high mobility group box-1 silence on diabetic retinopathy: An in vivo study. Int. J. Clin. Exp. Pathol..

[84] Liang W.J., Yang H.W., Liu H.N., Qian W., Chen X.L. (2020). HMGB1 upregulates NF-kB by inhibiting IKB-α and associates with diabetic retinopathy. Life Sci..

[85] Abu El-Asrar A.M., Alam K., Garcia-Ramirez M., Ahmad A., Siddiquei M.M., Mohammad G., Mousa A., De Hertogh G., Opdenakker G., Simó R. (2017). Association of HMGB1 with oxidative stress markers and regulators in PDR. Mol. Vis..

[86] El-Asrar A.M., Missotten L., Geboes K. (2011). Expression of high-mobility groups box-1/receptor for advanced glycation end products/osteopontin/early growth response-1 pathway in proliferative vitreoretinal epiretinal membranes. Mol. Vis..

[87] Abu El-Asrar A.M., Imtiaz Nawaz M., Kangave D., Siddiquei M.M., Geboes K. (2012). Osteopontin and other regulators of angiogenesis and fibrogenesis in the vitreous from patients with proliferative vitreoretinal disorders. Mediat. Inflamm..

[88] El-Asrar A.M., Nawaz M.I., Kangave D., Geboes K., Ola M.S., Ahmad S., Al-Shabrawey M. (2011). High-mobility group box-1 and biomarkers of inflammation in the vitreous from patients with proliferative diabetic retinopathy. Mol. Vis..

[89] Shen Y., Cao H., Chen F., Suo Y., Wang N., Xu X. (2020). A cross-sectional study of vitreous and serum high mobility group box-1 levels in proliferative diabetic retinopathy. Acta Ophthalmol..

[90] Abu El-Asrar A.M., Nawaz M.I., Siddiquei M.M., Al-Kharashi A.S., Kangave D., Mohammad G. (2013). High-mobility group box-1 induces decreased brain-derived neurotrophic factor-mediated neuroprotection in the diabetic retina. Mediat. Inflamm..

[91] Abu El-Asrar A.M., Nawaz M.I., Kangave D., Abouammoh M., Mohammad G. (2012). High-mobility group box-1 and endothelial cell angiogenic markers in the vitreous from patients with proliferative diabetic retinopathy. Mediat. Inflamm..

[92] Abu El-Asrar A.M., Siddiquei M.M., Nawaz M.I., Geboes K., Mohammad G. (2014). The proinflammatory cytokine high-mobility group box-1 mediates retinal neuropathy induced by diabetes. Mediat. Inflamm..

[93] Abu El-Asrar A.M., Mohammad G., Nawaz M.I., Siddiquei M.M. (2015). High-Mobility Group Box-1 Modulates the Expression of Inflammatory and Angiogenic Signaling Pathways in Diabetic Retina. Curr. Eye Res..

[94] Chang Y.C., Lin C.W., Hsieh M.C., Wu H.J., Wu W.S., Wu W.C., Kao Y.H. (2017). High mobility group B1 up-regulates angiogenic and fibrogenic factors in human retinal pigment epithelial ARPE-19 cells. Cell Signal..

[95] Surai P.F., Kochish I.I., Fisinin V.I., Kidd M.T. (2019). Antioxidant Defence Systems and Oxidative Stress in Poultry Biology: An Update. Antioxidants.

[96] Hayashi H., Kirakosyan A., Kaufman P.B. (2009). Molecular biology of secondary metabolism: Case study for *Glycyrrhiza* plants. Recent advances in plant biotechnology.

[97] Mollica L., De Marchis F., Spitaleri A., Dallacosta C., Pennacchini D., Zamai M., Agresti A., Trisciuoglio L., Musco G., Bianchi M.E. (2007). Glycyrrhizin Binds to High-Mobility Group Box 1 Protein and Inhibits Its Cytokine Activities. Chem. Biol..

[98] Shirazi Z., Aalami A., Tohidfar M., Sohani M.M. (2018). Metabolic Engineering of Glycyrrhizin Pathway by Over-Expression of Beta-amyrin 11-Oxidase in Transgenic Roots of Glycyrrhiza glabra. Mol. Biotechnol..

[99] Liu L., Jiang Y., Steinle J.J. (2019). Glycyrrhizin Protects the Diabetic Retina against Permeability, Neuronal, and Vascular Damage through Anti-Inflammatory Mechanisms. J. Clin. Med..

[100] Liu L., Jiang Y., Steinle J.J. (2017). Inhibition of HMGB1 protects the retina from ischemia-reperfusion, as well as reduces insulin resistance proteins. PLoS ONE.

[101] Liu L., Jiang Y., Steinle J.J. (2019). Epac1 and Glycyrrhizin Both Inhibit HMGB1 Levels to Reduce Diabetes-Induced Neuronal and Vascular Damage in the Mouse Retina. J. Clin. Med..

[102] Fukui M., Kitagawa Y., Nakamura N., Yoshikawa T. (2003). Glycyrrhizin and serum testosterone concentrations in male patients with type 2 diabetes. Diabetes Care.

[103] Selvam C., Mutisya D., Prakash S., Ranganna K., Thilagavathi R. (2017). Therapeutic potential of chemically modified siRNA: Recent trends. Chem. Biol. Drug Des..

[104] Bumcrot D., Manoharan M., Koteliansky V., Sah D.W. (2006). RNAi therapeutics: A potential new class of pharmaceutical drugs. Nat. Chem. Biol..

[105] Jiang S., Chen X. (2017). HMGB1 siRNA can reduce damage to retinal cells induced by high glucose in vitro and in vivo. Drug Des. Dev. Ther..

[106] Bralley E.E., Greenspan P., Hargrove J.L., Wicker L., Hartle D.K. (2008). Topical anti-inflammatory activity of Polygonum cuspidatum extract in the TPA model of mouse ear inflammation. J. Inflamm. (Lond.).

[107] Peng W., Qin R., Li X., Zhou H. (2013). Botany, phytochemistry, pharmacology, and potential application of Polygonum cuspidatum Sieb.et Zucc.: A review. J. Ethnopharmacol..

[108] Han J.H., Koh W., Lee H.J., Lee H.J., Lee E.O., Lee S.J., Khil J.H., Kim J.T., Jeong S.J., Kim S.H. (2012). Analgesic and anti-inflammatory effects of ethyl acetate fraction of Polygonum cuspidatum in experimental animals. Immunopharmacol. Immunotoxicol..

[109] Sohn E., Kim J., Kim C.S., Lee Y.M., Kim J.S. (2016). Extract of polygonum cuspidatum attenuates diabetic retinopathy by inhibiting the high-mobility group box-1 (HMGB1) signaling pathway in streptozotocin-induced diabetic rats. Nutrients.

[110] Zheng Y.Q., Wei W., Zhu L., Liu J.X. (2007). Effects and mechanisms of paeoniflorin, a bioactive glucoside from paeony root, on adjuvant arthritis in rats. Inflamm. Res..

[111] Jiang F., Zhao Y., Wang J., Wei S., Wei Z., Li R., Zhu Y., Sun Z., Xiao X. (2012). Comparative pharmacokinetic study of paeoniflorin and albiflorin after oral administration of Radix Paeoniae Rubra in normal rats and the acute cholestasis hepatitis rats. Fitoterapia.

[112] Iwahara N., Hisahara S., Kawamata J., Matsumura A., Yokokawa K., Saito T., Fujikura M., Manabe T., Suzuki H., Matsushita T. (2016). Role of suppressor of cytokine signaling 3 (SOCS3) in altering activated microglia phenotype in APPswe/PS1dE9 mice. J. Alzheimer’s Dis. JAD.

[113] Zhu S.H., Liu B.Q., Hao M.J., Fan Y.X., Qian C., Teng P., Zhou X.W., Hu L., Liu W.T., Yuan Z.L. (2017). Paeoniflorin Suppressed High Glucose-Induced Retinal Microglia MMP-9 Expression and Inflammatory Response via Inhibition of TLR4/NF-κB Pathway Through Upregulation of SOCS3 in Diabetic Retinopathy. Inflammation.

[114] Yan C., Ward P.A., Wang X., Gao H. (2013). Myeloid depletion of SOCS3 enhances LPS-induced acute lung injury through CCAAT/enhancer binding protein δ pathway. FASEB J..

[115] Vlachojannis J.E., Cameron M., Chrubasik S. (2009). A systematic review on the effectiveness of willow bark for musculoskeletal pain. Phytother. Res..

[116] Kong C.S., Kim K.H., Choi J.S., Kim J.E., Park C., Jeong J.W. (2014). Salicin, an extract from white willow bark, inhibits angiogenesis by blocking the ROS-ERK pathways. Phytother. Res..

[117] Ishikado A., Sono Y., Matsumoto M., Robida-Stubbs S., Okuno A., Goto M., King G.L., Keith Blackwell T., Makino T. (2013). Willow bark extract increases antioxidant enzymes and reduces oxidative stress through activation of Nrf2 in vascular endothelial cells and Caenorhabditis elegans. Free Radic. Biol. Med..

[118] Song Y., Tian X., Wang X., Feng H. (2019). Vascular protection of salicin on IL-1β-induced endothelial inflammatory response and damages in retinal endothelial cells. Artif. Cells Nanomed. Biotechnol..

[119] Fink M.P. (2007). Ethyl pyruvate: A novel anti-inflammatory agent. J. Intern. Med..

[120] Lee Y.M., Kim J., Jo K., Shin S.D., Kim C.S., Sohn E.J., Kim S.G., Kim J.S. (2013). Ethyl pyruvate inhibits retinal pathogenic neovascularization by downregulating HMGB1 expression. J. Diabetes Res..

[121] Maurer M., Bader M., Bas M., Bossi F., Cicardi M., Cugno M., Howarth P., Kaplan A., Kojda G., Leeb-Lundberg F. (2011). New topics in bradykinin research. Allergy.

[122] Zhu Y., Li X.Y., Wang J., Zhu Y.G. (2019). Bradykinin alleviates DR retinal endothelial injury by regulating HMGB-1/NF-κB pathway. Eur. Rev. Med. Pharmacol. Sci..

[123] Zhao L., Patel S.H., Pei J., Zhang K. (2013). Antagonizing Wnt pathway in diabetic retinopathy. Diabetes.

[124] Chao J., Bledsoe G., Chao L. (2016). Protective Role of Kallistatin in Vascular and Organ Injury. Hypertension.

[125] Ma J.X., King L.P., Yang Z., Crouch R.K., Chao L., Chao J. (1996). Kallistatin in human ocular tissues: Reduced levels in vitreous fluids from patients with diabetic retinopathy. Curr. Eye Res..

[126] Xing Q., Zhang G., Kang L., Wu J., Chen H., Liu G., Zhu R., Guan H., Lu P. (2017). The Suppression of Kallistatin on High-Glucose-Induced Proliferation of Retinal Endothelial Cells in Diabetic Retinopathy. Ophthalmic Res..

[127] Zhang Q., Guy K., Pagadala J., Jiang Y., Walker R.J., Liu L., Soderland C., Kern T.S., Ferry R., He H. (2012). Compound 49b prevents diabetes-induced apoptosis through increased IGFBP-3 levels. Investig. Ophthalmol. Vis. Sci..

[128] Jiang Y., Pagadala J., Miller D., Steinle J.J. (2013). Reduced insulin receptor signaling in retinal Müller cells cultured in high glucose. Mol. Vis..

[129] Berger E.A., Carion T.W., Jiang Y., Liu L., Chahine A., Walker R.J., Steinle J.J. (2016). β-Adrenergic receptor agonist, compound 49b, inhibits TLR4 signaling pathway in diabetic retina. Immunol. Cell Biol..

[130] Nebbioso M., Alisi L., Giovannetti F., Armentano M., Lambiase A. (2019). Eye drop emulsion containing 0.1% cyclosporin (1 mg/mL) for the treatment of severe vernal keratoconjunctivitis: An evidence-based review and place in therapy. Clin. Ophthalmol..

[131] Wang P., Chen F., Zhang X. (2020). Cyclosporine-a attenuates retinal inflammation by inhibiting HMGB-1 formation in rats with type 2 diabetes mellitus. BMC Pharmacol. Toxicol..

[132] Carmo A., Cunha-Vaz J.G., Carvalho A.P., Lopes M.C. (2000). Effect of cyclosporin-A on the blood-retinal barrier permeability in streptozotocin-induced diabetes. Mediat. Inflamm..

